# Newcastle Disease Virus Vectored Bivalent Vaccine against Virulent Infectious Bursal Disease and Newcastle Disease of Chickens

**DOI:** 10.3390/vaccines5040031

**Published:** 2017-09-26

**Authors:** Sohini Dey, Madhan Mohan Chellappa, Dinesh C. Pathak, Satish Gaikwad, Kalpana Yadav, Saravanan Ramakrishnan, Vikram N. Vakharia

**Affiliations:** 1 Recombinant DNA Laboratory, Division of Veterinary Biotechnology, Indian Veterinary Research Institute, Izatnagar 243 122, UP, India; dennisdinesh16@gmail.com (D.C.P.); satish.bty@gmail.com (S.G.); kalpanayadav33@gmail.com (K.Y.); 2 Immunology Section, Indian Veterinary Research Institute, Izatnagar 243 122, UP, India; dearsaromib@yahoo.com; 3 Institute of Marine and Environmental Technology, University of Maryland Baltimore County, Baltimore, MD 21202, USA; vakharia@umbc.edu

**Keywords:** Newcastle disease virus vector, IBDV-VP2 protein, bivalent vaccine candidate, humoral and cell mediated immune responses, protection efficacy

## Abstract

Newcastle disease virus (NDV) strain F is a lentogenic vaccine strain used for primary vaccination in day-old chickens against Newcastle disease (ND) in India and Southeast Asian countries. Recombinant NDV-F virus and another recombinant NDV harboring the major capsid protein VP2 gene of a very virulent infectious bursal disease virus (IBDV); namely rNDV-F and rNDV-F/VP2, respectively, were generated using the NDV F strain. The rNDV-F/VP2 virus was slightly attenuated, as compared to the rNDV-F virus, as evidenced from the mean death time and intracerebral pathogenicity index analysis. This result indicates that rNDV-F/VP2 behaves as a lentogenic virus and it is stable even after 10 serial passages in embryonated chicken eggs. When chickens were vaccinated with the rNDV F/VP2, it induced both humoral and cell mediated immunity, and was able to confer complete protection against very virulent IBDV challenge and 80% protection against virulent NDV challenge. These results suggest that rNDV-F could be an effective and inherently safe vaccine vector. Here, we demonstrate that a bivalent NDV-IBDV vaccine candidate generated by reverse genetics method is safe, efficacious and cost-effective, which will greatly aid the poultry industry in developing countries.

## 1. Introduction

In India, Newcastle disease (ND) and infectious bursal disease (IBD) have been a constant threat to the poultry industry [1,2] which causes heavy economic losses in terms of mortality and immunosuppression in young chicks due to ND and IBD, respectively. Infections caused by Newcastle disease virus (NDV) may exacerbate infections with other etiological agents, and reduce the chicken’s ability to respond to vaccination. ND is a highly contagious and fatal viral disease affecting all species of birds, and so does IBD which is an immunosuppressive disease of immature chickens caused by a birnavirus, infectious bursal disease virus (IBDV). The actively dividing and differentiating lymphocytes of the B-cell lineage of the bursa of Fabricius of particularly young chickens get affected, resulting in morbidity, mortality and immune-suppression along with ineffective responses to vaccines. IBDV belongs to the Avibirnavirus genus of the Birnaviridae family, and has non-enveloped, icosahedral capsid consisting of double-stranded RNA segments, namely segment A and B [3]. The larger ORF1 of segment A encodes for a 110-kDa polyprotein which auto-catalytically splices into mature viral proteins; VP2 (48 kDa), VP3 (33–35 kDa) and VP4 (24 kDa) [4]. VP2 is the major capsid protein that carries the immunogenic determinants and the major host-protective antigen, able to elicit neutralizing antibodies [5]. Very virulent strain of IBDV (vvIBDV) has emerged in the late 1980’s [6] and the virus is continuously evolving in the field with changes in antigenicity and virulence. Currently, the disease is controlled by live attenuated or inactivated IBDV vaccines which may not give complete protection against vvIBDV strain [7]. The inactivated or killed viruses are usually given to birds in pre-laying stage to induce higher levels of antibody production for at least 2 weeks. The commercially available live vaccines based on classical virulent strains induce both cellular and humoral immunity but may also show necrosis and lymphoid depletion. They may show reversion to virulence and vaccine associated reactions, resulting in clinical disease and production losses. Non-replicating antigens such as inactivated whole viruses, viral subunits or recombinant viral antigens are not efficacious unless combined with adjuvants and administered repeatedly [8].

Newcastle disease virus (NDV), an avian paramyxovirus-1 is being developed as a vaccine vector candidate. NDV, strain F is a virus of low virulence originally reported in England [9]. Since then, in several countries in Europe, Africa and Asia, the use of this virus as an immunizing agent in the form of a live vaccine has been studied. However, the complete genome of this virus was not determined to be made amenable for its usage as a vector. Therefore, we elucidated the complete genome sequence of this NDV strain F [10], which allowed us to generate NDV in the laboratory from cDNAs with predefined genetic markers by reverse genetics technology. In this study, for the first time we have developed a reverse genetics system exclusively for the NDV vaccine strain F, incorporating the major capsid protein gene VP2 of a very virulent IBDV to generate a bivalent vaccine candidate. Previously, two bivalent vaccine candidates, utilizing NDV backbone with LaSota strain and a chimeric NDV LaSota virus with the L gene of clone-30, were generated expressing the VP2 gene of IBDV. The investigators reported that VP2 protein was not incorporated into the virions of recombinant virus [11] and only the humoral immune responses in susceptible birds were evaluated in protection against these diseases [11,12]. Moreover, none of these investigators could unequivocally show the expression of VP2 protein by Western blot analysis using purified recombinant NDV and demonstrate protective cell mediated immune response. Therefore to overcome these pitfalls, we added the hemagglutinin (HN) signal sequence at the N-terminal of VP2 and generated a recombinant rNDV-F/VP2 virus, using reverse genetics. In this study, we evaluated the efficacy of NDV-vectored IBDV vaccine in affording protection against both NDV and vvIBDV challenges and the vaccine candidate induced both humoral and cellular immune responses.

## 2. Material and Methods

### 2.1. Virus and Cells

Very virulent IBDV strain, available at Indian Veterinary Research Institute, India was grown in eleven-days-old specific-pathogen-free (SPF) embryonated chicken eggs inoculated through the chorio-allantoic membrane (CAM) route. The plaque purified F strain seed virus, available with the viral repository of Indian Veterinary Research Institute, Izatnagar was propagated in eleven-days-old SPF embryonated chicken eggs via the allantoic route. The viral antigen was prepared and purified by ultracentrifugation procedure using sucrose gradient, as described [13]. Vero cells were used for virus recovery and propagation. The cells were grown at 37 °C in the minimum essential medium containing 10% fetal bovine serum. Exogenous trypsin in the form of 10% allantoic fluid was added to Vero cells for viral rescue and subsequent passages of the virus in Vero cells.

### 2.2. Construction of Full-Length Clone of NDV, Strain F

Viral RNA was extracted from the purified virus using TRIzol (Sigma, St. Louis, MO, USA), according to manufacturer’s instructions. Reverse transcription was carried out with the extracted RNA using the Superscript RT kit (Invitrogen, Carlsbad, CA, USA) to synthesize the first strand cDNA. Oligonucleotide primers were synthesized for amplifying the entire genome of NDV-F virus as overlapping fragments and the complete genome was elucidated [10] and deposited in the GenBank (Accession No. KC987036.1). A full-length cDNA copy of the RNA genome was constructed by assembling three overlapping synthetic fragments (GenScript, Piscataway, NJ, USA) by standard cloning techniques. The fragments were ligated serially by natural or artificially created unique restriction sites. Two ribozyme sites were incorporated at both the ends of the full-length clone of the virus, namely hammerhead ribozyme (HHRz) at the 5′ end and hepatitis delta virus ribozyme (HdvRz) at the 3′ end. The HHRz sequence (5′-CTGATGAGTCCGTGAGGACGAAACTATAGGAAAGGAATTCCTATAGTC-3′) and HdvRzsequence (5′GGGTCGGCATGGCATCTCCACCTCCTCGCGGTCCGACCTGGGCATCCGAAGGAGGACAGACGTCCACTCGGATGGCTAAGGGAGAGCCA-3′) were fused with the first and third fragment by overlapping PCR [14].

**Figure 1:**
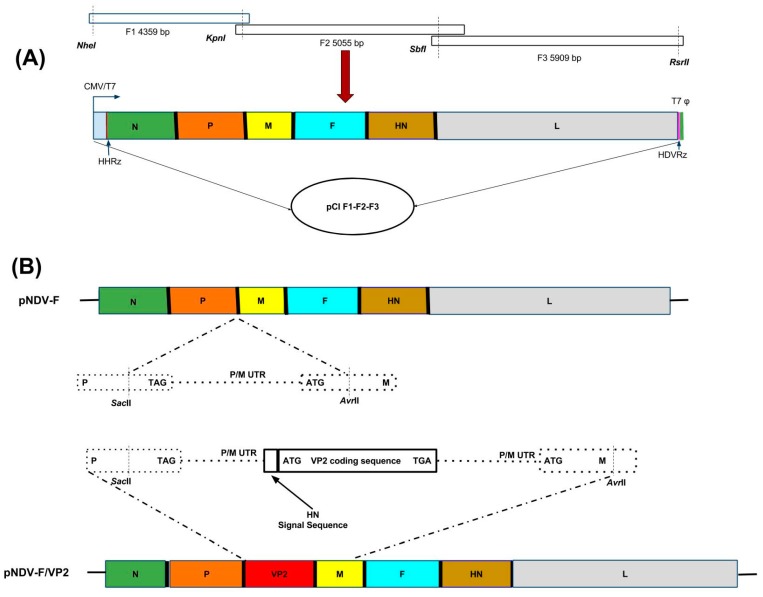
Generation of full-length clone of strain F with and without VP2 gene cassette. (**A**) Schematic representation of the construction of full-length clone pCI F1-F2-F3 of Newcastle disease virus (NDV) strain F. Assembly was carried out by sequential cloning of F1, F2 and F3 fragments into pCI vector. Fragment F1 contains hammerhead ribozyme (HHRz) at the 5′-end and fragment F3 contains hepatitis delta ribozyme (HdvRz) at the 3′-end. Restriction sites used for cloning are indicated. Abbreviations: CMV/T7, cytomegalovirus immediate-early enhancer and promoter/T7 RNA polymerase promoter; T7ɸ, T7 transcription termination. (**B**) Schematic representation of the NDV genome with the SacII and AvrII sites between the P and M genes and insertion of VP2 gene with HN signal between these sites.

Three plasmids, namely F1, F2 and F3 with a size of 4359 bp, 5055 bp and 5909 bp, respectively and representing the entire genome complement of NDV strain ‘F’, were synthesized in pUC57 based vector having the NDV sequence (GenScript, USA). The restriction enzyme sites SacII (1661 bp position), AvrII (1736 bp position), RsrII (2662 bp position), NheI (10,168 bp and 14,530 bp positions), and AvrII (14,866 bp position) were deleted in the F1, F2 and F3 synthesized fragments. To assemble the full length clone, the F1 fragment was double digested with NheI and KpnI restriction enzymes and cloned between NheI and KpnI sites of the modified pCI vector harboring the HHRz and HdvRz sequences [15]. Second, the F2 fragment was double digested with KpnI and RsrII restriction enzymes and cloned in the F1 pCI vector between KpnI site of F1 and RsrII sites of the Hdv sequence and named as F1-F2 pCI vector. Third, the F3 fragment was double digested with SbfI and RsrII enzymes and cloned into F1-F2 pCI vector and named as pCI F1-F2-F3. This entire cassette F1-F2-F3 inserted into the modified mammalian expression vector pCI (Promega, Madison, WI, USA) (Figure 1A) was under the control of the cytomegalovirus promoter [15].

The IBDV VP2 gene cassette was synthesized by incorporating the haemagglutinin neuraminidase (HN) signal sequence (141 bp) of NDV to the 5′-end of the VP2 sequence (1326 bp) and the cassette is flanked by the restriction sites SacII and AvrII; NDV P gene coding (cds); P gene non-coding (NC); intergenic sequence (IGS); M gene NC; and P gene NC; IGS; M gene NC; M cds. A termination codon (TGA) was placed at the end of the VP2 gene. Four additional nucleotides TAAA were added after the termination codon to maintain the “rule of six”. The amplified fragment was digested with SacII and AvrII enzymes, then inserted into the P–M intergenic region at nucleotide position 2354 and 5251 of the NDV genome (Figure 1B). The generated plasmid was designated as pNDV-F/VP2.

### 2.3. Construction of Supporting Plasmids

Using the full-length clone of pNDV-F/VP2 as template, the open reading frames (ORFs) of NP (1469 bp), P (1187 bp), and L (6614 bp) genes were amplified by PCR. A Kozak consensus sequence, GCCACC, was incorporated in front of the start codon of each ORF [16]. The NP and P ORFs were cloned between EcoRI and NotI restriction sites; whereas L ORF was cloned between NheI and NotI restriction sites of the pCI vector (Promega, USA) by digesting with appropriate restriction enzymes. The pNP, pP, and pL support plasmids were sequenced using an automated DNA Sequencer, as described earlier. All these plasmids were under the control of cytomegalovirus promoter (CMV).

### 2.4. Rescue of Recombinant NDV-F/VP2 Virus in Vero Cells

Transfection was done using the full-length clone of the virus along with the support plasmids using standard protocols. Briefly, the Vero cells were grown upto 80% confluency before transfection. The media was removed from the cells and the cells were incubated with OptiMEM media for 1 h. The plasmids pNDV-F and/or pNDV-F/VP2 (5 µg), pCI-NP (2.5 µg), pCI-P (1.5 µg) and pCI-L (0.5 µg) were diluted in 500 µL OptiMEM medium to which Lipofectamine LTX reagent (Invitrogen, USA) was added and incubated for 30 min. The OptiMEM media was removed from the Vero cells and the plasmid-lipofectamine mixture was added onto the cells in a drop wise manner. One mL of OptiMEM was added to the cells and was left undisturbed at 37 °C incubator with 5% CO_2_ for 24 h. After 24 h, the transfected mixture was removed and the cells were incubated with M199 medium (Life Technologies, Carlsbad, CA, USA) with 10% allantoic fluid obtained from SPF embryonated chicken eggs. The cells were left undisturbed for 72 h. The cells were then freeze-thawed thrice and re-infected onto healthy Vero cells. The process was repeated thrice.

### 2.5. Confirmation of Virus Stability

The recombinant viruses namely, rNDV-F and rNDV-F/VP2 were passaged serially in SPF embryonated chicken eggs ten times and was analyzed for its stability. RNA from the recombinant virus present in the allantoic fluid was isolated by TRIzol reagent according to standard protocols. A reverse transcription polymerase chain reaction (RT-PCR) was set up with different sets of primers to confirm the presence of different regions of NDV and the VP2 gene of IBDV.

To confirm the presence of the IBDV VP2 gene cassette in the full-length NDV genome, RT-PCR was done using the forward primer (nt position 3064 at P cds of the viral genome) and the reverse primer with respect to the 3′-end of VP2 gene (1693 bp). The PCR product was cloned into a T/A cloning vector and the plasmid DNA was sequenced.

### 2.6. Characterization of Recombinant Viruses

The reactivity of the NDV proteins and the VP2 protein in the purified recombinant virus, obtained by sucrose gradient centrifugation procedure [13], was assessed using anti-NDV antibody (Abcam, Cambridge, MA, USA) and mouse anti-VP2 monoclonal antibody (Abcam, USA), respectively by Western blotting.

Expression of the VP2 protein in rNDV-F/VP2 virus was examined by IFA using IBDV-specific monoclonal antibody (mAb) against the VP2 protein (Abcam, USA; 1:100) and an NDV-specific polyclonal antibody (Abcam, USA; 1:50). Briefly, confluent monolayers of Vero cells were infected with recombinant viruses at a multiplicity of infection (MOI) of 0.01. After 24 h, the infected cells and control cells were washed with phosphate-buffered saline (PBS) and fixed with 4% paraformaldehyde (Affymetrix Inc., Cleveland, OH, USA) for 1 h at room temperature, followed by addition of 0.5% Triton X-100 (Sigma, USA) to permeabilize the cells at room temperature for 10 min. The permeabilized cells were blocked with 5% bovine serum albumin (Sigma, USA) for 30 min at 37 °C. After blocking, the cells were incubated for 1 h with a mixture of antibodies. Cells were washed with PBS and incubated with a mixture of the FITC labeled sheep anti-mouse IgG (Sigma, USA, 1:250 dilution) and Alexa Fluor(R) 568-labeled goat anti-chicken IgG (Thermo Fisher Scientific, Carlsbad, CA, USA, 1:200 dilutions) for 1 h at 37 °C. Nuclear staining was carried out using DAPI (Thermo Fisher Scientific, USA, 1:200 dilutions). Fluorescence images were photographed using a FluoViewFV1000 confocal microscope (Olympus, Waltham, MA, USA) with matching excitation/emission filters for FITC and Alexa Fluor 568.

The recombinant viruses were further characterized by inoculating eleven-days-old SPF embryonated chicken eggs and incubating at 37 °C. The embryos were observed for 96 h and at the end of incubation period, the lesions were evaluated.

The growth kinetics of the parent and the recombinant virus in tissue culture was analyzed using a multistep growth curve analysis. Monolayer of Vero cells (in triplicate) were infected with each virus at a multiplicity of infection (MOI) of 0.01 and supernatant was collected and replaced with an equal volume of fresh medium at 12 h interval until 72 h. The viral titers of these samples were determined by Reed and Muench method [17].

### 2.7. Titration of Recombinant NDV-F Virus by Real Time PCR

A fragment of 113 bp of F gene of NDV was amplified using the primer pairs (Forward: 5′- TAA GCT CCT CCC GAA TCT-3′ at position 4732–4749 and reverse: 5′-TAC GGA TAG AGT CAC CAA GG-3′ at position 4825–4844 of the NDV viral genome) and the PCR product was cloned in the T/A vector. The OD value of the recombinant plasmid DNA concentrations was measured at 260 nm/280 nm (600 ng/µL) and plasmid copy number was calculated using the formula described by Adams, 2006. The standard plasmid DNA was diluted 10-fold serially from 10^−1^ to 10^−12^ and was used for making the standard curve along with the sample cDNA. The real-time PCR was performed on CFX96 real time system (Bio-Rad, Hercules, CA, USA) in duplicate for each sample. The reaction mixture was prepared in a total volume of 20 µL consisting of 2 µL cDNA, 10 µL of QuantiTect SYBR Green Master Mix (Qiagen, Hilden, Germany), and primers 0.5 µL each. Real time PCR was carried out with the following program: 1 cycle at 95 °C for 5 min, followed by 40 cycles of 94 °C for 30 s, 60 °C for 45 s, 70 °C for 45 s and 1 cycle of 94 °C for 30 s. The final step was to obtain a melt curve for the PCR products to determine the specificity of the amplicons.

### 2.8. Biological Characterization of the Recombinant Viruses

The recombinant viruses were characterized biologically by mean death time (MDT) and intra cerebral pathogenicity index (ICPI) analysis. The MDT analysis was carried out in eleven-days-old embryonated chicken eggs and ICPI was carried out in one-day-old SPF chickens, according to standard procedures [18].

### 2.9. Vaccine Efficacy Testing in Chickens

#### 2.9.1. Dose Optimization and Safety Test

A total of 42 day-old SPF chicks were randomly allocated to seven groups. The first group was kept as control; the remaining groups were vaccinated intranasally with rNDV-F and rNDV-F/VP2 recombinant viruses at different doses viz., 10^5^ EID_50_/bird, 10^6^ EID_50_/bird and 10^7^ EID_50_/bird, respectively. Blood was collected for serum analysis from all the birds at days 0, 7, 14, 21 days post-immunization. The antibody response was checked by ELISA and cellular immune response was checked by antigen specific lympho-proliferative assay using 3-(4,5-dimethylthiazol-2-yl)-2,5-diphenyltetrazolium bromide (MTT) dye. Following dose optimization, a 10-fold dose of the optimized dose (10^7^ EID_50_/bird) of recombinant viruses were given intranasally to 10 SPF chickens and observed for 21 days for any abnormality.

#### 2.9.2. Immunization Trial

The immunization and protection studies of the recombinant viruses were evaluated in SPF chicks maintained under standard management practices at biosafety level 2 where chicks were provided with autoclaved feed and water. A total of ninety SPF day-old chicks were distributed randomly in 7 groups as follows: unvaccinated (no challenge), unvaccinated (IBDV and NDV challenge, 10 birds in each group), rNDV-F/VP2 virus (20 birds), rNDV-F virus (10 birds), commercially available NDV ‘F’ (RDF-Ranikhet disease ‘F’ strain), killed and live IBDV vaccines (10 birds in each group). At one day of age, the birds were vaccinated with rNDV-F/VP2 virus and rNDV-F virus through the intra-nasal route at a dose of 10^6^ EID_50_/bird and the same dose was repeated two weeks post primary vaccination;RDF, killed and live IBDV vaccines were vaccinated with the recommended dose and route. The unvaccinated groups were given PBS. Two weeks following booster immunization, 10 chickens in each group were subdivided into two factions: control, rNDV-F/VP2 virus, rNDV-F virus and commercially available RDF groups, which were challenged with 10^5^ ELD_50_ virulent NDV through intramuscular route (i/m), and the second faction: control, rNDV-F/VP2 virus, killed and live IBDV vaccines groups that were challenged with 10^3^ ELD_50_ very virulent IBDV strain through intra-ocular route. All birds were observed daily for two weeks for any clinical signs and mortality.

#### 2.9.3. Determination of Protection from Challenge

Protection against IBDV challenge was assessed by studying the occurrence of mortality, presence of viral antigen in bursa four days post-challenge as determined by agar gel precipitation test, bursal gross lesions, and bursa to body weight ratio (bursa/body weight B/B%) ten days post-challenge. Clinical signs and mortality were observed up to a period of 14 days post-challenge. The dead and surviving chickens were sacrificed and bursa of Fabricius was collected for gross and histological examinations with haematoxylin and eosin (H&E) staining. The bursae from all the groups were examined histologically to determine the severity of bursal damage. The bursal lesions were graded according to a scale of 0 to 4: (0) no lesions; (1) mild scattered cell depletion in a few follicles; (2) moderate with 1/31/2 of the follicle atrophied or with depleted cells; (3) diffuse with atrophy in all follicles; and (4) acute inflammation and acute necrosis, typical of IBDV infection. Protection against NDV was assessed for two weeks post-challenge for the presence of clinical signs and mortality.

#### 2.9.4. Determination of Humoral Immune Response by ELISA and HI Assay

Briefly, 96-well ELISA plates were coated with recombinant VP2 antigen of IBDV or recombinant nucleoprotein (NP) antigen of NDV, diluted in the coating buffer (100 mM bicarbonate buffer, pH 9.0) at 4 °C overnight and blocked with 2% bovine serum albumin (BSA). The sera samples, diluted 1:200 in PBS were added on to the plate and incubated at 37 °C for 1 h. The relative antibody titers were calculated by sample to positive (S/P) ratio and end point titer was determined, as described previously [19]. Serum samples with an end point titer of more than 500 and 200 were considered positive for IBD and ND, respectively. HI was performed to analyze the NDV antibody titers as per standard protocol.

### 2.10. Determination of Cellular Immune Response

#### 2.10.1. Lymphocyte Proliferation Assay

At 28 days post immunization (dpi), peripheral blood mononuclear cells (PBMCs) were collected from both vaccinated and unvaccinated control groups (n = 6) by using ficoll-hypaque (specific gravity 1.077 g/mL) (Sigma, St. Louis, MO, USA). The viability of cells was checked by trypan blue dye exclusion assay. The cell number was adjusted to be at 1 × 10^7^ cells/mL. One hundred microliters of medium with cells were added in triplicates to the cell culture plates and stimulated with recombinant VP2 protein at a concentration of 5 µg/mL, and virulent NDV at a concentration of 10 µg/mL; ConA (10 µg/mL) was used as positive control along with non-stimulated control. The plates were incubated at 37 °C in 5% CO_2_ atmosphere with 95% relative humidity. After 60 h, 20 µL of MTT dye (5 mg/mL) was added to all the wells and incubated for further 4 h. One hundred microliters of DMSO was added to all the wells and mixed to dissolve the formazan crystals. Spectrophotometric reading was taken at 570 nm and stimulation index (S.I) was calculated as the ratio between mean OD value of stimulated cells and the mean OD value of the non-stimulated control.

#### 2.10.2. Flow Cytometric Analysis

At 14 and 21 dpi, PBMCs were collected from the vaccinated and control groups (n = 6) and analyzed by flow cytometry. Briefly, 1 × 10^5^ cells were taken in a microcentrifuge tube to which 1:10 dilution of anti-chicken CD3, CD4 and CD8 monoclonal antibodies (mAb) (Serotec, Oxford, UK) were added. The tubes were incubated in the dark at room temperature for 30 min. The cells were then washed twice with PBS to remove the unbound mAb molecules and analyzed in a FACS Calibur Flow Cytometer (BD Biosciences, San Jose, CA, USA) to estimate the percentage of CD4 and CD8 cells in the sample.

### 2.11. Statistical Analysis

SPSS 20.0 statistical software was used to analyze the results. One-way ANOVA was employed to evaluate the differences in the mean values of various parameters and Waller-Duncan as post-hoc test.

## 3. Result

### 3.1. Generation of Full-Length Clone of Strain F with and without VP2 Gene Cassette

The full-length clone of NDV F (pNDV-F) was assembled in pCI vector from three synthetic overlapping clones, F1, F2 and F3, as shown in Figure 1A. In generating the IBDV VP2 gene cassette, the full-length VP2 gene of IBDV was inserted between the non-coding regions of the P and M genes of the full length clone with a size of 2913 bp, incorporating the necessary start and stop signals from NDV and the signal sequence of HN of NDV at the 5′ end of VP2 to express and incorporate the VP2 in NDV (Figure 1B). The total size of the pNDV-F/VP2 was precisely 16,872 bp, which followed the ‘rule of six’.

### 3.2. Rescue of Recombinant NDV with and without IBDV VP2 Gene Cassette from Cloned cDNA

Vero cells were used to rescue the recombinant viruses after transfecting the cells with the full-length NDV clones, along with the support plasmids. After three blind passages in the Vero cells, the supernatant was inoculated into the allantoic cavity of nine-days-old SPF embryonated chicken eggs. The viral cytopathic effect (CPE) produced in the Vero cells typically consisted of clumping and rounding of cells that were observed by 48 h post-infection (hpi) and fusion of cells with syncytia formation as observed 72 hpi under the light microscope. The sequence data of the rescued virus confirmed that the junctions of the NDV with respect to the IBDV gene in the transcription cassette were intact confirming the recombinant nature of the virus generated by reverse genetics. The genetic stability of the recombinant viruses was confirmed by sequencing the viral RNA after 10 passages in embryonated chicken eggs. The sequencing results indicated that the integrity of the VP2 gene and its expression was preserved even after 10 serial egg passages.

### 3.3. Biological and Molecular Characterization of the Recombinant Viruses

**Figure 2:**
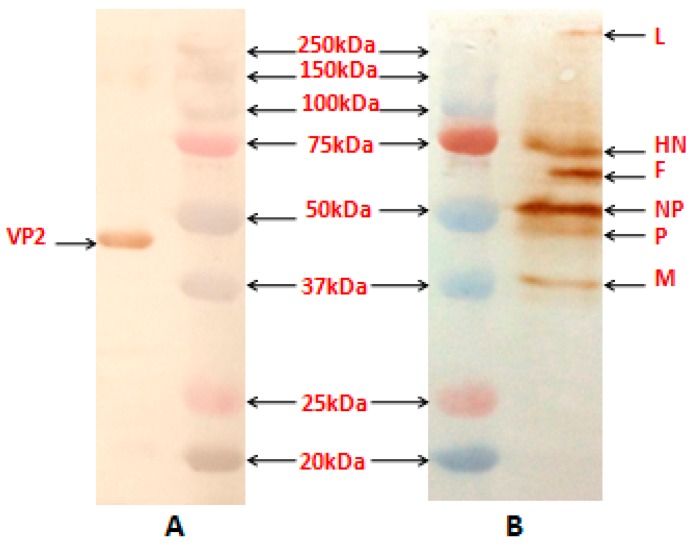
Western blot analysis of the proteins produced by purified recombinant NDV containing VP2 gene of infectious bursal disease virus (IBDV). Purified virus samples were separated on 12.5% sodium dodecyl sulfate polyacrylamide gel electrophoresis (SDS-PAGE). Proteins were then transferred to nitrocellulose membrane and blocked in 1% BSA in TBST. Membranes were incubated with either mouse anti-VP2 monoclonal antibody or chicken anti-NDV polyclonal antibody at 4 °C for 1 h. Membranes were washed in TBST and then incubated with secondary antibody conjugated with HRP in 1% BSA in TBST for 1 h at room temperature. Membranes were washed in TBST and incubated with Sigma Fast DAB (Sigma, USA) color development reagent for 15 min and then visualized. (**A**) The position of the VP2 protein of about 48-kDa (right) and marker proteins (Precision Plus Protein Standards, Bio-Rad, USA) are as indicated. The blot was probed with anti-mouse IBDV-VP2 monoclonal antibody (Abcam, USA). (**B**) The positions of the NDV L, HN, F, NP, P and M (right) and marker proteins are as indicated. The blot was probed with anti-chicken NDV polyclonal antibody.

**Figure 3:**
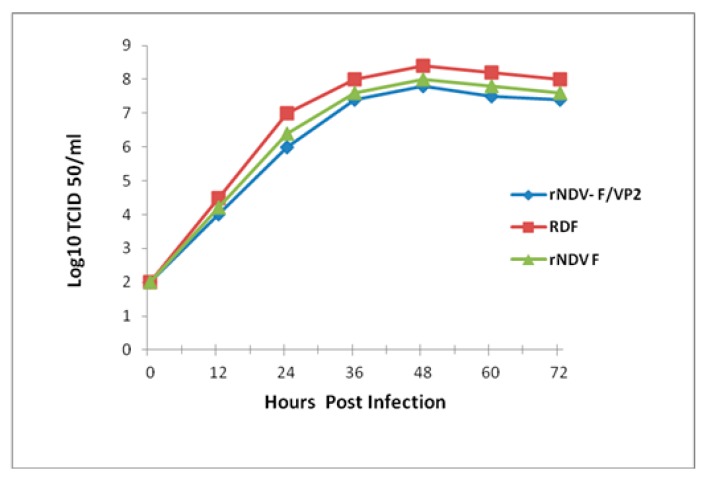
Multicycle growth kinetics of parent NDV strain F and recombinant viruses rNDV-F/VP2 and rNDVF in Vero cells. Monolayer of Vero cells were infected with each of the viruses at a multiplicity of infection (MOI) of 0.01 and supernatant collected and replaced with an equal volume of fresh medium at 12 h interval until 72 h, and viral titers were determined by limiting dilution assay and calculated as TCID_50_ by Reed and Muench method.

**Figure 4:**
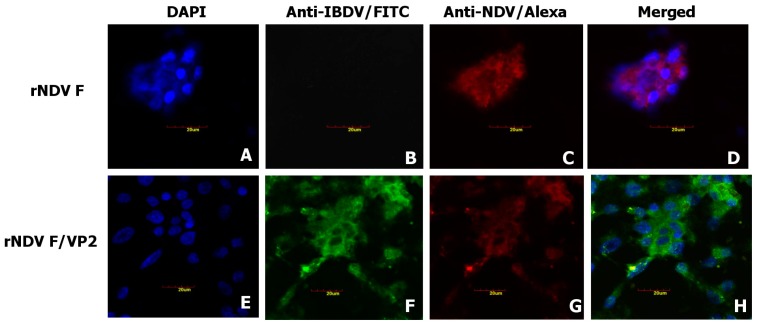
Immunofluorescence analysis of VP2 protein expression in rNDV-F/VP2 virus. Immunofluorescence analysis of VP2 protein expression in rNDV-F/VP2 virus. Confluent monolayers of Vero cells were infected with recombinant viruses rNDV-F/VP2 and rNDV-F at a multiplicity of infection (MOI) of 0.01. The cells were examined by immunofluorescence assay (IFA) with IBDV specific mAb against the VP2 protein (Abcam, USA) and an NDV-specific polyclonal antibody (Abcam, USA), followed by a mixture of the FITC labeled sheep anti-mouse IgG (B,F) and Alexa Fluor 568-labeled goat anti-chicken IgG (C,G). Fluorescence was monitored and photographed using a confocal microscope (Olympus FV 10, USA) at a magnification of 60× under UV light of matching excitation filters for FITC and Alexa Fluor 568 respectively.

The biological properties of RDF, rNDV-F and rNDV-F/VP2 include a mean death time of 164 h, 172 h and 184 h, respectively and intracerebral pathogenicity index value of 0.1, 0.1 and 0.0, respectively. The embryos were observed to have mild haemorrhages and normal growth, which was typical of a lentogenic virus. An RT-PCR was set up with known sets of primers to amplify the NDV genome sequences, as well as the VP2 gene sequence. The presence of VP2 gene in the recombinant virus was confirmed by sequencing the RT-PCR product obtained using gene-specific primers with an amplicon size of 1326 bp. The sequence data showed that the junction of the NDV with respect to the VP2 gene in the transcription cassette was intact (1693 bp), confirming that recombinant virus contained the VP2 gene. A single protein band of 48-kDa was detected in the Western blot when the purified rNDV-F/VP2 virus was reacted with VP2 monoclonal antibody (Abcam, USA), indicating the incorporation of VP2 protein in the recombinant virus (Figure 2A). Western blot analysis also confirmed the presence of purified NDV, as all the viral proteins had reactivity with the NDV polyclonal antibody (Figure 2B). The multistep growth kinetics study was carried out on Vero cells with a collection interval of 12 h until 72 h of infection (Figure 3). The parent and recombinant viruses had a similar kinetic and replicative efficiency as evident by the growth curve. There was a mild reduction in the titers of the recombinant viruses as compared to the parent virus during the entire period of study. The expression of VP2 protein in rNDV-F/VP2 infected cells was observed as a bright green fluorescence when anti-IBD mAb was used in immunofluorescence assay (IFA). Further, the presence of NDV was observed as a bright red fluorescence in infected cells when anti-NDV polyclonal antibody was used, as shown in Figure 4.

At 48 h post-inoculation, the maximum titers for rNDV-F, rNDV-F/VP2 and RDF were 10^8^ TCID_50_/mL, 10^7.8^ TCID_50_/mL and 10^8.4^ TCID^50^/mL, respectively. The recombinant virus was titrated by real-time PCR using partial F gene cloned into T/A vector and used as the standard. The standard curve was plotted using the log copy numbers in x-axis and the CT values in y-axis and the equation derived is as follows, Y = −3.34321429x + 40.56073508. The quantitative real time PCR efficiency was 99.12%. The copy number of the recombinant virus was compared with the copy number of parent vaccine strain F with a known EID_50_ to derive EID_50_ of the recombinant virus. The titer of the virus determined by this method was 10^7.96^ EID_50_/mL.

### 3.4. Immunization and Protective Efficacy Studies and Challenge with very Virulent IBDV

Humoral and cellular immune responses were induced in SPF birds that were inoculated with rNDV-F and rNDV-F/VP2 at a dose of 10^6^ EID_50_/bird. All the birds were active and alert. There was no abnormal local or systemic reaction observed and the birds were apparently normal at a ten-fold optimized dose of 10^7^ EID_50_/bird.

#### 3.4.1. Humoral Immune Response

**Figure 5:**
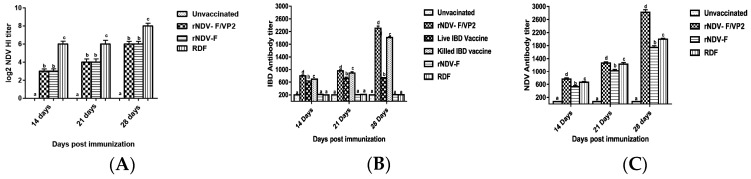
NDV and IBDV specific serum antibody responses in the experimental chickens. (**A**) HI assay was carried out 14 and 28 days post-immunization. All HI titers are expressed as mean reciprocal log2 titre ± SEM (standard error of the mean) (n = 10). Statistical differences were calculated by one-way ANOVA with *p* < 0.01 and Waller-Duncan as post-hoc test. The antibody titers as determined on 14, 21 and 28 days post-immunization by ELISA, (**B**) higher than 500 were considered positive for IBDV antibody and (**C**) higher than 200 were considered positive for NDV. Data represent the mean ± standard error. Statistical analysis was done by one-way ANOVA (*p* < 0.01 and Waller-Duncan as post-hoc test. Level not connected by same letter are significantly different (*p* < 0.01).

Blood samples were collected from the birds in each experimental group on 0, 14, 21 and 28 dpi and serum was separated and the NDV and IBDV antibody titers were determined by HI test and ELISA. The sera collected from the vaccinated birds at 14, 21 and 28 dpi showed significant antibody response (*p* < 0.01) against NDV as well as IBDV in comparison to control. At 14, 21 and 28 dpi, HI titers against NDV was highest in birds immunized with RDF vaccine. However, the groups vaccinated with rNDV-F and rNDV-F/VP2 showed higher ELISA titers than the birds given commercial NDV vaccine against NDV (Figure 5A,B). The antibody titers in the rNDV-F/VP2 vaccinated birds against IBDV were significantly higher (*p* < 0.01) than that of the live as well as inactivated vaccine groups (Figure 5C). All the sera samples from the control groups were negative.

#### 3.4.2. Cellular Immune Response

##### Lymphocyte Proliferation Assay

**Figure 6:**
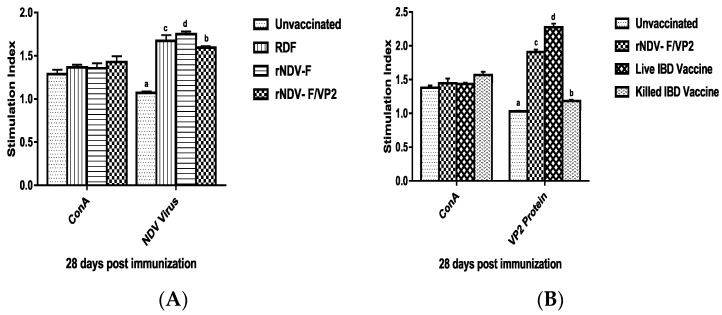
Antigen specific lymphocyte proliferative response in chickens on 28 days post immunization. Chicken PBMCs from immunized and control groups (n = 6) were stimulated with NDV virus (**A**), recombinant VP2 protein expressed in *Saccharomyces cerevisiae* (**B**), and concanavalin A (conA) as positive control. Lymphocyte proliferative response was measured and expressed as stimulation index. All the data presented as mean value ± standard error and Waller-Duncan as post-hoc test. Level not connected by same letter are significantly different (*p* < 0.01).

Lymphocyte proliferation assay using MTT dye was performed at 28 days dpi to assess the cellular immune response. PBMCs from all the experimental group of birds showed proliferation on stimulation with ConA, indicating the normal healthy immune status of the birds. In case of in vitro stimulation with NDV, the recombinant NDV-F vaccine group showed the maximum SI followed by commercial RDF vaccine and rNDV-F/VP2 groups (Figure 6A). On in vitro stimulation with recombinant VP2 protein expressed in *Saccharomyces cerevisiae*, the commercial live IBD vaccine group showed the maximum SI followed by rNDV-F/VP2 and killed IBD vaccine groups on 28 dpi (*p* < 0.01) (Figure 6B).

##### Flow Cytometric Analysis

**Figure 7:**
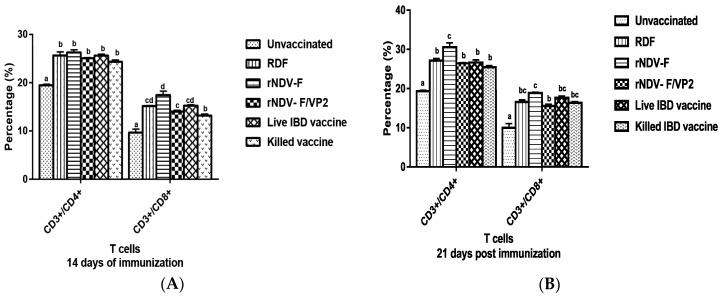
Flow cytometric analysis of CD4^+^ and CD8^+^ T cells in peripheral blood mono-nuclear cells (PBMCs) of vaccinated and unvaccinated birds at 14 (**A**) and 21 (**B**) dpi. PBMCs were collected from the birds and 1 × 10^5^ cells were analyzed after addition of chicken specific Mabs conjugated with fluorescent dyes. Data represent the percentage of cells ± SEM. The values depicted in different lowercase superscripts differ significantly (*p* < 0.01) and Waller-Duncan as post-hoc test.

On 14 and 21 dpi, PBMCs from both vaccinated and control groups were analyzed for CD4, CD8 T cell subsets. Birds from vaccinated groups showed significantly (*p* < 0.01) higher CD4 T cells than the unvaccinated group. On 14 and 21 dpi, the birds from rNDV-F group showed the highest percentage of CD4 T cells as well as CD8 T cells, followed by RDF vaccine, live IBD vaccine groups and rNDV-F/VP2 vaccine groups (Figure 7).

#### 3.4.3. Protection Studies with the Bivalent Vaccine Candidate against IBDV and NDV Challenge

The protection efficacy as evaluated by studying the occurrence of mortality in susceptible chickens, bursal gross lesion score, B/B ratios and percent of protection is being summarized in Table 1. The birds injected with the commercial IBDV live vaccine, rNDV-F/VP2 groups showed no mortality, whereas the IBDV inactivated vaccine group showed 10% and unvaccinated IBDV challenged group showed 100% mortality. The B/B ratios in the vaccinated groups were comparable with that of the normal uninfected control group and significantly (*p* < 0.01) higher than the control challenge group. The percentage of virus clearance as determined by agar gel precipitation test showed 90% clearance in rNDV-F/VP2 and live vaccine groups, and 83.33% clearance in killed vaccine group. Bursal lesion score was the highest in the unvaccinated challenge group. In case of vaccinated groups, the score was the highest in killed vaccine group followed by rNDV-F/VP2 and live vaccine groups. Histopathologically, the bursa of birds from challenge group showed extensive haemorrhages, necrosis and severe depletion of lymphoid cells typical of IBD infection. Bursa from killed and live vaccine groups showed follicular atrophy in some places, mild to moderate depletion of lymphoid cells and mild necrosis. In rNDV-F/VP2 group, mild depletion of lymphoid cells with intact follicular structure was observed.

The protective efficacy as evaluated by the rNDV-F or rNDV-F/VP2 viruses following inoculation of NDV challenge virus through intramuscular route resulted in 80% protection in the vaccinated SPF birds. Birds were observed for 14 days post-challenge. All the birds in the unvaccinated control group showed clinical signs and succumbed to infection between 4 and 6 days post challenge. Affected birds became paralyzed in a day after commencement of clinical signs. However, the onset of clinical signs in birds belonging to rNDV-F and rNDV-F/VP2 groups was late and with less severe clinical signs as compared to the control groups.

## 4. Discussion

In this study, we have generated a recombinant NDV-F virus (rNDV- F) with an incorporated IBDV VP2 gene from a very virulent IBDV strain into it (rNDV-F/VP2), using a low virulent NDV-F strain to be used as a vaccine vector. The rNDV-F/VP2 was evaluated as a bivalent vaccine candidate against ND and IBD in SPF chickens. Currently, lentogenic NDV strains Hitchner B1, Lasota, F and mesogenic R2B strains are widely used as live vaccines in India [10]. The strain F vaccine is usually used in young chickens but is also suitable for use as a vaccine in chickens of all ages. As a viral vector, NDV strain LaSota has been widely used to express glycoproteins of avian metapneumovirus subgroup C [20], glycoprotein gB and gD genes of infectious laryngotracheitis [21,22], HA of H5 avian influenza [23], VP2 gene of IBDV [11,12]. Besides poultry viral diseases, LaSota as a viral vector has been used to express rabies glycoprotein [24], haemagglutinin (H) or fusion protein (F) of canine distemper [25]. In this study, we have reported for the first time generation of a recombinant NDV ‘F’ strain viral vector expressing the VP2 capsid gene of vvIBDV, which has the ability to induce both the arms of immune responses. It has been reported earlier that the LaSota strain often causes post-vaccination respiratory signs and it could be used as a booster vaccine in flocks vaccinated with F or B1 [26]. However, the recombinant virus generated with NDV strain F with IBDV VP2 was found to be safe, stable and able to show protective efficacy against virulent NDV and very virulent IBDV challenges. In most of the earlier reports, researchers have utilized glycoproteins as foreign antigens to be expressed from NDV viral vector. VP2 protein of IBDV is a non-glycosylated capsid protein of IBDV. The absence of appropriate signal in VP2 protein to direct them to golgi apparatus prompted us to utilize the HN signal from NDV to be fused with the VP2 gene for it to be incorporated into the virion of rNDV-F/VP2 virus. Hence, to obtain the actual virus titer, a real time PCR assay was performed by cloning a portion of F gene into a T/A cloning vector and used as a standard. This incorporation of VP2 was evidenced by an induction of sufficient immune responses in SPF chickens. The rNDV-F/VP2 virus was slightly attenuated, as compared to the rNDV-F virus, may be due to the increase in size of the full-length NDV F genome after insertion of the foreign protein VP2 gene, as reported earlier by several groups [27,28]. The results showed that the rNDV-F or rNDV-F/VP2 were safe and no abnormal clinical signs and death were observed in chickens even at 10-fold higher than the recommended dose. This vaccine would be more acceptable for vaccination in young chicks considering the earlier reports that the lentogenic LaSota produces mild respiratory symptoms in young chickens.

IBDV is very resistant in the environment and the commonly used sanitizers are not sufficient to prevent the infection. Thus, vaccination plays an important role in the prevention of the disease. Different modified live vaccines (MLVs) have been developed and classified as mild intermediate, intermediate plus IBD vaccines depending upon their ability to break through the interference of maternally derived antibodies. However, MLVs are not fully efficacious against IBDV field challenges and induce moderate to severe bursal lesions and immunosuppression. The major capsid protein VP2 has proved to be an important target for generating cellular and humoral immune responses capable of conferring active and passive protection against IBDV infection [29,30]. There are several reports using recombinant live viral vectors including herpes virus of turkey [31,32], NDV [11], fowl pox virus [33], Marek’s disease virus [34] and avian adenovirus [35] as expression vectors incorporating VP2 with the aim of protecting birds against IBD. Humoral immunity plays an important role in the clearance of IBD following vaccination with rapid infiltration of T cells into the bursa and upregulation of CMI related genes following infection with very virulent IBD led to the hypothesis that CMI responses are also crucial for protection against IBDV [36]. Similarly, CMI plays an important role in protection of chickens vaccinated against NDV and contributes to viral clearance [37]. Although humoral antibody remains the primary mechanism in affording protection against virulent NDV or IBDV, CMI responses whose quantification are labor intensive is considered equally important in the field challenges [38]. The present study reports the induction of both humoral and cell mediated immune responses by the recombinant virus rNDV-F/VP2 against NDV and IBDV. The subsets of T lymphocytes, including cytokine-secreting CD4^+^ T helper cells (MHC class II) and CD8^+^ cytotoxic T lymphocytes (MHC class I) as presented in the host post vaccination are the principal cells of the CMI response. The birds vaccinated with the recombinant viruses showed a significantly higher CD4^+^ and CD8^+^ T lymphocytes as compared to the commercial vaccines against IBDV and NDV in Flow cytometric analysis. The results were supported by the lymphocyte transformation test, wherein the rNDV-F and rNDV-F/VP2 induced antigen specific lymphocyte proliferative response when stimulated with virulent NDV virus or VP2 protein respectively. The recombinant viruses were extremely effective in eliciting antibody mediated immune reactions beside cell mediated reactions, as indicated by production of high titers of antigen specific antibodies. In this study, the HI antibody titers could attain 64 with the rNDV-F or rNDV-F/VP2 against NDV which was 2 log2 titer less than the parental vaccine RDF at 28 days post vaccination. HI titers of 32 or higher are protective against NDV infection [39]. However, the recombinant virus could provide only 80% protection against NDV challenge. In a similar study, recombinant fowl pox virus expressing NDV fusion and haemagglutinin genes and ILTV gB gene induced detectable ELISA antibody titres against NDV and ILTV but failed to elicit significant HI titers against NDV [40].

As the number of vaccine candidates developed using NDV reverse genetics are increasing every year, researchers are able to decipher the differences in the immune responses accorded by these recombinant viruses. It has been noted that all the recombinant NDV vaccines are not equal in their ability to replicate in chickens. Hence, each vaccine that is created should be evaluated for its ability to replicate in chickens, and to induce a protective immune response against a virulent NDV challenge [41]. However, significantly higher levels of antibody titers were noticed in NDV and IBDV ELISA of the rNDV-F/VP2 group reflecting the presence of higher levels of neutralizing antibodies against both NDV and IBDV, proving that the recombinant virus could elicit a strong humoral response. Further on vvIBDV challenge, none of the vaccinated birds showed clinical signs or mortality and the control unvaccinated birds started showing symptoms from 3rd day post challenge. All the dead birds showed gross bursal lesions, haemorrhages in the thigh muscles typical of IBD. The vaccinated groups showed significantly less bursal lesions with mild depletion of lymphocytes and intact follicular structure.

## 5. Conclusions

A reverse genetics system based on NDV strain ‘F’ was developed and two recombinant viruses namely rNDV-F and rNDV-F/VP2 were rescued in Vero cells. Both the viruses were slightly attenuated as compared to the parent NDV-F virus and on immunization trials, the findings showed that the recombinant lentogenic NDV strain F is an avirulent, safe viral vector for VP2 of IBDV and could completely protect the chickens against very virulent IBDV. The developed vaccine (rNDV-F/VP2) induced humoral as well as cellular immune responses against both NDV and IBDV. The bivalent vaccine candidate generated by reverse genetics against NDV and IBDV will greatly aid the poultry industry in developing countries in reducing the cost of the vaccine.

## Figures and Tables

**Table 1:** Protection efficacy in different groups of chickens against vvIBDV challenge.

Sl. No	Groups	^a^ Mortality	^b^ B/B Ratio	^c^ Bursal Lesion Score	^d^ Protection Percentage
1	Unvaccinated unchallenged	-	0.67 ± 0.28	0 ± 0	-
2	Unvaccinated challenged	10/10 (100%)	0.24 ±0.09	4.6 ± 0.2	0/10 (0%)
3	Live IBDV vaccine	0/10 (0%)	0.48 ± 0.01	2.2 ± 0.2	10/10 (100%)
4	Inactivated IBDV killed vaccine	1/10 (10%)	0.43 ± 0.02	2.8 ± 0.2	9/10 (90%)
5	rNDV F/VP2	0/10 (0%)	0.54 ±0.02	2.0 ± 0.2	10/10 (100%)

^a^ Mortality was recorded during 10 days period after virus challenge and presented as number of dead chickens/total number of chickens in each group and percentage in parenthesis. ^b^ Bursa/Body weight ratios was calculated by bursal weight divided by body weight × 100 and presented as the mean ± standard deviation from each group. ^c^ Bursal lesion score was measured from 0 to 4 based on the increasing severity of bursal lesions; (0) no lesions, (1) mild scattered cell depletion in a few follicles, (2) moderate with 1/3–1/2 of the follicles atrophied or with depleted cells, (3) diffuse with atrophy in all follicles, (4) acute inflammation and necrosis typical of IBD and expressed as the mean ± standard deviation from each group. ^d^ Protection percentage was determined as the number of protected chickens/total number of chickens in a group ×100.
